# A Strain-Based Intelligent Tire to Detect Contact Patch Features for Complex Maneuvers

**DOI:** 10.3390/s20061750

**Published:** 2020-03-21

**Authors:** Mª Fernanda Mendoza-Petit, Daniel García-Pozuelo, Vicente Díaz, Oluremi Olatunbosun

**Affiliations:** 1 Mechanical Engineering Department, Universidad Carlos III de Madrid, Avd. De la Universidad, 28911 Madrid, Spain; dgramos@ing.uc3m.es (D.G.-P.); vdiaz@ing.uc3m.es (V.D.); 2 School of Mechanical Engineering, University of Birmingham, Edgbaston B15 2TT, UK; o.a.olatunbosun@bham.ac.uk

**Keywords:** strain gauge, sensors, intelligent tire, effective radius, contact length, fuzzy logic system, simulation, wheel speed

## Abstract

Tires are essential components of vehicles and are able to transmit traction and braking forces to the contact patch, contribute to directional stability, and also help to absorb shocks. If these components can provide information related to the tire–road interaction, vehicle safety can be increased. This research is focused on developing the tire as an active sensor capable to provide its functional parameters. Therefore, in this work, we studied strain-based measurements on the contact patch to develop an algorithm to compute the wheel velocity at the contact point, the effective rolling radius and the contact length on dynamic situations. These parameters directly influence the dynamics of wheel behavior which nowadays is not clearly defined. Herein, hypotheses have been assumed based on previous studies to develop the algorithm. The results expose to view an experimental test regarding influence of the tire operational condition (slip angle, vertical load, and rolling velocity) onto the computed parameters. This information is used to feed a fuzzy logic system capable of estimating the effective radius and contact length. Furthermore, a verification process has been carried out using CarSim simulation software to get the inputs for the fuzzy logic system at complex maneuvers.

## 1. Introduction

The tire is the only component in contact with the road surface. It is a flexible structure covered commonly with rubber compounds, and when it is rolling onto a surface, it causes the phenomena of hysteric losses due to its periodical deformation in the contact patch. Thus, the knowledge of the tire dynamic parameters helps to understand the mechanics of the interaction between the frictional surfaces [1,2]. This tire–road interaction is directly related to the sliding condition in the tire footprint, hence, this information can be used to estimate the dynamic friction available to transmit the vehicle’s traction [3]. The accurate control of the forces between tire and road is the main goal for the automotive industry in order to enhance vehicles’ maneuverability and safety.

For providing the driver with better means of vehicle control, the tire is facing a new challenge as an active sensor. There is an increasing demand for a whole working program of intelligent tire systems [4,5,6,7]. Therefore, an intelligent tire must be able to monitor the tire parameters related to a vehicle’s dynamic behavior, e.g., load transfer, tangential forces, tire condition, road conditions or friction coefficient, the velocity at the contact patch, contact length, and effective rolling radius, which currently, are not commonly known. The better the study and measurement of the behavior of the tire, the easier it is to control the working parameters that change that behavior in order to ensure the safety of the vehicle.

In order to achieve an optimization of the current vehicle dynamic control systems, a considerable amount of literature suggests the potential of intelligent tire systems [8,9,10,11,12]. The Intelligent or smart tire relies on sensors embedded in the tire. Among the technologies developed in this context are: Energy Harvesting Technologies (EHTs) used by adapting a harvester unit to the tire vibration spectra [13,14,15,16]; Micro Electro Mechanical Systems (MEMSs) consisting of a piezoresistive n-polysilicon strain sensors of a thin membrane that serves to amplify the strain in the wafer [17]; Surface Acoustic Wave (SAW) sensors consisting of delay lines formed by two reflectors with different distances from the interdigital transducer (IDT) to measure the tire pressure and temperature parameters automatically [18,19]; Fiber Bragg Grating (FBG)-based strain measurements, which is a multi-sensing device to measure the circumferential strain [20,21]; Optical Sensors, which are commonly used to measure the deformations of the carcass under different in-plane tire forces [22,23,24]; Tri-axial (MEMS) accelerometers, which have been placed inside tires for friction force estimations [25,26,27]. However, it is difficult to extract characteristics of interest from the signal of these MEMS due to its sensitivity to the noise generated from the road surface [12]. As can be inferred from all these technologies applied on smart tires, several approaches have been proposed. Nevertheless, strain gauges are gaining attention due to their low cost, robustness and high reliability in their measurements. Furthermore, their reduced dimensions and low weight avoid additional load on the tire tread, and the measured values are not affected by the rotation of the wheel [12]. Commonly, this type of sensor is attached to in the inner surface of the tire, avoiding damage. The most recent studies related to intelligent tires demonstrate the correlation of the strain gauges measurements with the tire operational condition [12,28,29,30,31,32,33,34,35,36]. Another perspective to estimate the tire parameters uses virtual sensors based on vehicle dynamics models [9,37,38,39,40,41]; however, many simplifications must to be made resulting in notable error and are not accurate enough for all driving conditions.

Among the dynamic parameters to monitor in a tire, it has been observed that in most of the literature related to the tire strain measurements, the contact length is pointed as an indicator of the peak-to-peak distance through the strain rate curve. However, none of them achieve real-time computational value from the strain measurement while the tire rolls over a surface [11,21,25,34]. A similar situation occurs regarding the effective radius and the velocity at the contact patch. The effective radius represents the relation between the linear speed in the contact patch and the angular speed of the wheel. This parameter is directly related to the sliding occurring in the contact patch. Further, only a few of them show the effect of varying the tire working conditions on the behavior of these parameters [25,35]. Nonetheless, these parameters have an enormous importance due to being closely related to the vehicles’ traction conditions, which involves the adhesion-slip in the tire–road contact, the control of emissions, the comfort, the maneuverability, the damping effects, and the loss of vehicle control [2,9,37,42,43]. Monitoring of the contact patch length, effective rolling radius, and velocity provides a better description of the phenomenon occurring in the tire contact patch. A considerable amount of literature has suggested their estimation as an innovative approach to enhance the functionality of the different active control systems attending as benefits. Yang et al. [34] showed that the strain measurements in steady straight-line condition provides information related to rolling speed which affects tire lock-up and skidding of Anti-lock Braking Systems (ABS) and the vertical load to warning about overloading and damage to dampers. Matsuzaki and Todoroki [36] suggested the possibility of developing a warning system for tire–road conditions using strain gauges for the purpose of enhancing the performance of ABS. Through estimating of the real-time frictional forces and velocity of the tire, it is possible to estimate the road surface friction [44]. Cheli et al. [45] demonstrated that monitoring the tire friction without delays helps to prevent the earlier lock-up of the wheel and also reduce the stopping distance using the ABS, in the Electronic Braking Distribution (EBD) systems. It was detected that monitoring the vertical load yields an enhancement in the choice of the braking distribution curve.

The effective rolling radius estimation proposed by Tannoury et al. [40] requires to extract the traction and velocity from ABS; nonetheless, the main idea is getting the tire paraments to optimize the control systems, and not the reverse; a similar study was proposed by Carlson et al. [41]. Other types of studies are based on finite element models to derive relationships between the strain sensors and the braking torque, effective radius and contact length [36] or correlate tire working conditions (angular velocity, preload, and inflation pressure, braking/traction force and cornering force) with strain data [33]. Also based on a flexible ring model, the strain wave curve is fitted to estimate the tire dynamic parameters [11,46], but this approach does not include frictional effects [11]. An interesting experimental study is developed by Aguilar et al. [47] where the tire rolls on a set of large strain gauges installed on a drum’s surface. Hence, the strain gauges on the surface measured the interaction with the tire contact patch. The study identified two different zones, the one in full contact and the other in partial contact. These are related to the distribution of the vertical load on the contact patch. This observation is comparable to the results of strain gauges measurement on the tire inner liner which shows similar effects in the strain curve (maximum tensile-compressive values) [48].

This paper seeks to address how to monitor the tire parameters (contact patch length, effective rolling radius, and velocity at the contact patch) developing a practical real-time implementation using only the strain measurements and without requiring a high computational complexity. The proposed methodology is based on a strain-based tire data in order to describe the phenomenon occurring in the tire contact patch. Mendoza et al. [28] proposed a methodology to estimate all the frictional forces, the vertical load, and the slip angle in the tire contact patch through strain-based methods; the analysis made to the strain data also enabled the validation of the estimator in dynamic maneuvers. Therefore, this work represents another step in this research line [28,29,30,31,32,33] in order to be able to estimate the friction available on the tire–road contact surface, this being one of the main objectives in the development of an intelligent tire.

The most remarkable results to emerge from the present paper are the relations found between the estimated parameters and the operational conditions of the tire. Further tests carried out with CarSim concurred with the coherence in the initial findings. The proposed methodology is independent of the tire size, but the size of the sensor (strain gauge) should be selected according to the tire size in order to improve the simulation of the strain measurement of a “virtual bristle”; nonetheless, this has to be checked in more detail.

## 2. Materials and Methods

In this section, we explain the equipment and the instrumentation used to collect the strain-based tire measurement. Further, the section shows the tire operational condition used in the experimental data acquisition. The approach implemented in this work allows computing the contact patch length, the effective rolling radius, and the velocity at the contact patch from the measured strain data in order to obtain the maximum quantity of information from the instrumented tire.

### 2.1. Tire Testing System

The prototype of the intelligent Strain-based Intelligent Tire was tested in the Vehicle Dynamics Laboratory of the University of Birmingham.

**Figure 1:**
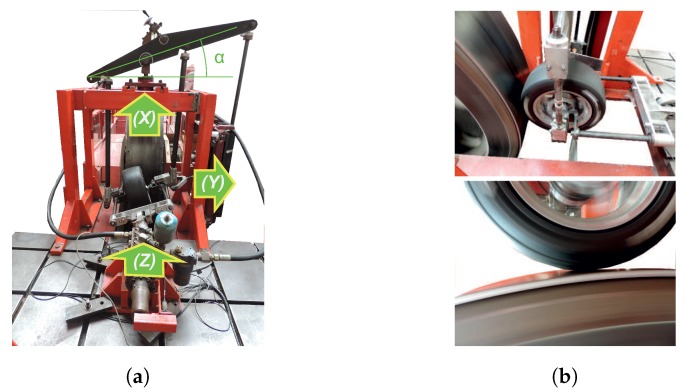
Test procedure applied to the intelligent tire: (**a**) The coordinate system used on the indoor tire test rig; (**b**) Instrumented tire set up on the tire test rig and its contact with the drum’s surface.

The experimental measurements were carried out in an indoor tri-axial tire test rig at the Vehicle Dynamics Laboratory, University of Birmingham. Figure 1a shows the axis coordinate system fixed on the test rig and, it also pointed out the instrument used to change the slip angle of the tire. The instrumented tire makes contact with the drum’s surface and, its vertical load is controlled by the actuation of a hydraulic cylinder on it in the *Z* direction, as is shown in Figure 1b. As an advantage, this sort of test rig allows accurate control of the tire’s operational conditions. The tire test rig is formed by a drum with a large diameter (2440 mm). The effect of the drum ’s curved surface introduces a small average error in the tire contact patch, being proper to approximate it as a flat contact surface [31,47]. The test rig has a group of actuators which can vary the tire position against the drum.

The tire testing system allows the drum’s velocity to be varied, the vertical load applied to the tire, and also can be controlled the slip angle and the camber angle of the instrumented tire.

### 2.2. Strain-Based Intelligent Tire Prototype

The tests were carried out with an instrumented tire mounted on the testing system. A slick radial tire DUNLOP SP SPORT 175/505 R13 (tubeless) for SAE Formula Student (FS) was used. Its unloaded radius ($R_{o}$) is 252.5 mm. The strain gauges were placed on the inner liner of the tire tread band and, they were fixed with the adhesive indicated by the strain gauges’ manufacturer. The tire deformations in the contact region were registered by the strain data.

**Figure 2:**
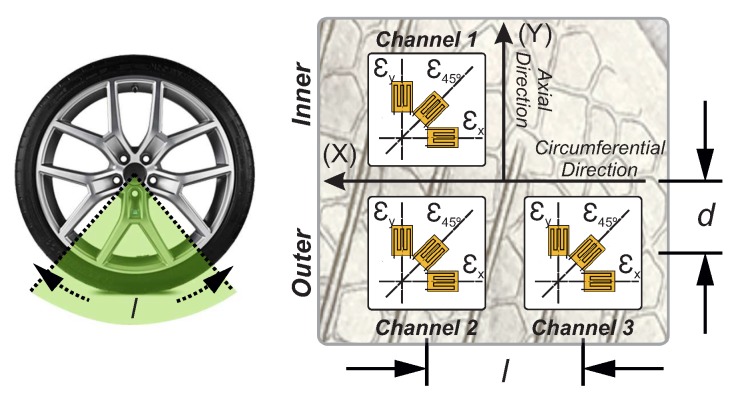
Disposition of the multiaxial gauge strain [28].

The strain gauges were placed on the inner liner of the tire tread band as shown in Figure 2. It is formed by three rectangular rosette strain gauges attached to the tire inner surface in longitudinal and lateral directions. Two of them are located in the same cross-section at the external side and the third one, at the inner side, of the tire tread. The distances “*d*” and “*l*” are about 40 mm and 515 mm, respectively. The strain gauge’s length is 2 mm.

Each strain gauge is connected to a channel to measure deformations in the circumferential and axial directions of the wheel plane. Two of them measure the axial deformations ($\mu \varepsilon_{y1}$, $\mu \varepsilon_{y3}$—channel 1 and channel 3, respectively) located symmetrically concerning the tread centerline and the other channel registered the circumferential strain ($\mu \varepsilon_{x}$—channel 2). The gauge resistance is 120 Ω and the resolution provided by the strain measurement is 0.001μϵ.

**Figure 3:**
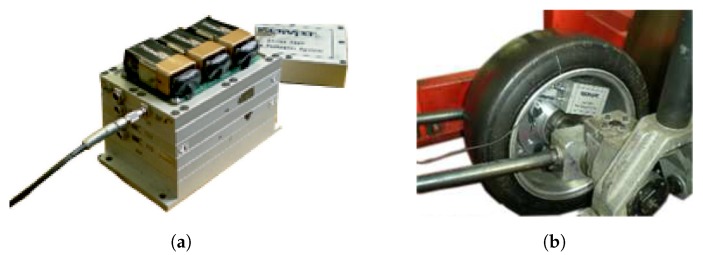
Acquisition system SoMat^®^ 2000: (**a**) Microprocessor for the data acquisition, and a Power/Communication module; (**b**) Data acquisition module installed on the tire [28].

The strain sensors are connected to a data acquisition module to measure strain at a sampling frequency of 1000 Hz. The portable equipment, SoMat^®^ 2000 Field Computer (Somat Corporation, Urbana, IL, USA ) was used as data acquisition system. Formed by a Wheatstone strain bridge, the data acquisition was configured in a quarter bridge. The hardware has a microprocessor for the data acquisition, and a Power/Communication module, equipped with batteries. The software used by this equipment is the TestPoint^®^ software (Capital Equipment Corporation, Norton, MA, USA) for Windows (WinTCS). The working range of the SoMat^®^ 2000 strain gauge module used covered from −5000μϵ to 5000μϵ. Figure 3 shows the hardware used for the data acquisition of the tire strains during the tests. Figure 3a shows the microprocessor and the power/communication module and, Figure 3b illustrates the microprocessor incorporated in the tire.

### 2.3. Test Conditions

During the test, the test conditions were varied to determine their influence on the dynamic behavior of the tire. The tire working conditions at the assays are into these bounds:

- Tire inflation pressure: 0.8 bar–1.4 bar. Step size 0.2 bar.
- Tire preload: 250 N–1000 N. Step size 250 N.
- Tire slip angle: 0°–10°. Step size 2°.
- Tire camber angle: 0°.

The test conditions were kept constant in terms of temperature and humidity (23° and 50% HR), as per the strain gauge manufacturer recommendations. Therefore, the effect of temperature changes on the tire or the inflation gas are not considered. Previous studies yielded the features of the data acquisition system to instrument the tire, also explaining the factors that affect it (sensors location, number of channels, frequency, memory capacity, among others) [31,34].

## 3. Algorithm to Compute Tire Parameters

The tire working conditions define the behavior of the frictional forces in the contact patch. The effective rolling radius, the contact length, and the velocity at the contact patch are dynamic parameters of the tire that influence the frictional forces transmitted by the tire. It is a critical aspect to guarantee road safety. Currently, there are studies where the relationships between tire deformation and contact length are analysed; also, between tire deformation and effective radius [12,35,36,48], but none of them provide real-time values in order to monitor these parameters.

This work proposes an algorithm to estimate the effective radius, the contact length, and velocity in the tire contact patch. The following abbreviations have been used to denote each of these parameters $cl_{w}$, $R_{eff}$, $V_{w}$. To achieve this goal herein has been used experimental data provided by the instrumented tire in the circumferential direction. In the experimental tests, the data is obtained under controlled conditions. Therefore, the drum’s speed, the vertical load, and the slip angle of the tire were varied to represent different rolling conditions.

The fundamentals used to develop this algorithm have been culled from [8,11,12,22,27,33,34,35,36,48,49]. The studied authors’ view let us propose hypotheses to compute these parameters from the strain time history for each working condition.

Once the proposed methodology is applied to the experimental strain-based data, the estimated tire parameters show variations under different controlled working conditions, giving information about their relationships.

**Figure 4:**
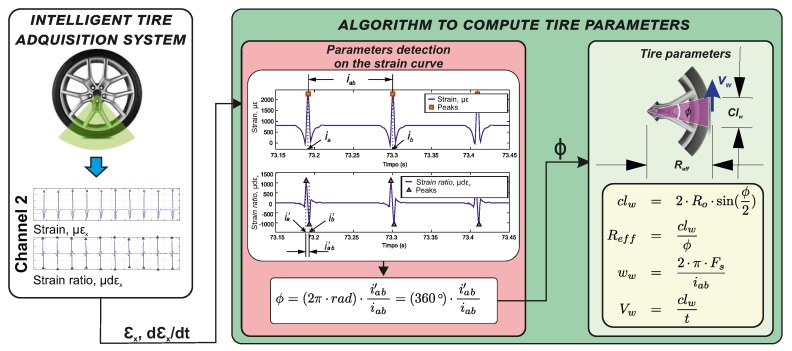
Workflow for the development of the intelligent tire detection algorithm.

To facilitate the understanding of the work done, Figure 4 shows the workflow to implement the proposed methodology to estimate the parameters of the tire in real-time conditions. The module of *intelligent tire acquisition system* describes the process to acquire strain measurements in the circumferential direction and calculate its time rate. The module *Algorithm to compute tire parameters* is split into two sections, the first one denoted as *Parameters detection on the strain curve* explains the selection of the points of interest on the strain and strain ratio curve and also the estimation of the central angle (Φ); , in the other section, *Tire parameters*, the tire parameters are computed through the equations of $cl_{w}$, $R_{eff}$, $V_{w}$ as shown. This process is explained in detail in Section 3.2.

Among the areas in which this study makes an original contribution are: a better understanding of the deformation curves, facilitating the calculation of the tire parameters ($cl_{w}$, $R_{eff}$, $V_{w}$) from the deformations measured on the tread, obtaining the magnitude of these parameters under controlled operating conditions, analysing the influence of the operating conditions on the estimated parameters, develop an estimator of $cl_{w}$ and $R_{eff}$ from the most influential variables, and verifying the estimator under different working conditions. The theoretical foundations used to develop the estimation algorithm of $cl_{w}$, $R_{eff}$, $V_{w}$ are described below.

### 3.1. Fundamentals of the Tire Strain Features Selection

This section studies the deformation process to which a point on the tire tread is subjected when it comes into contact with the road surface. Different theoretical foundations are used to develop the algorithm for estimating the tire parameters ($R_{eff}$, $cl_{w}$ and $V_{w}$) implemented in this work.

**Figure 5:**
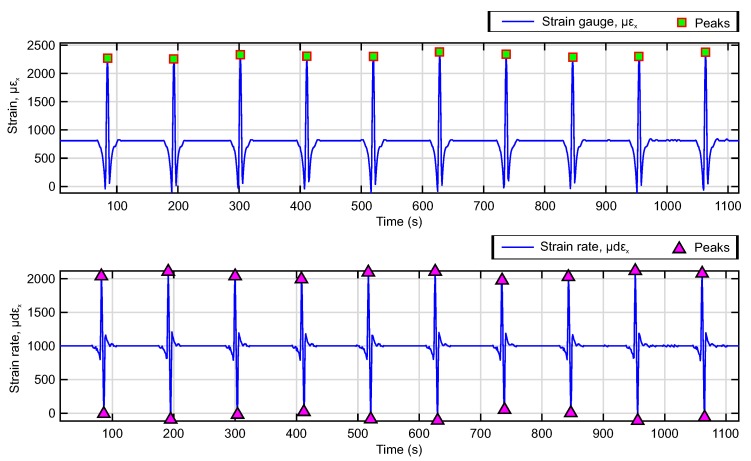
Selection of the deformation characteristics in the time history of the deformation in the circumferential direction ($\varepsilon_{x}$), and its derivative curve ($d\varepsilon_{x}$) Operating conditions: 0.8 bar, 0°, 50 km/h, 500 N.

Morinaga et al. [48] analysed the strain measured in the contact patch, emphasising the importance of understanding the meaning of the strain waveform. Based on their study, the selection of parameters is set at the maximum and minimum peaks of the circumferential strain curves and their time derivative. To extract these points, a maximum and minimum peak detection algorithm has been performed using MATLAB software. An example of the operation of the peak detection algorithm on the circumferential strain curve and its derivative curve is shown in Figure 5.

In this study, peak detection is performed to extract the length of the time interval or cycle between the maximum deformation peaks of the circumferential deformation curve, while, from the derivative curve, the length of the time interval between the maximum peak and the minimum peak of each cycle is extracted. These intervals, on the time axis, are the data required to implement the methodology proposed in this study.

The process of detecting strain parameters is carried out for each experimental condition. The experimental data enables the calculation of the effective radius, contact length, and velocity in the tire’s contact patch under controlled conditions. As a result, it is possible to assess the effects of the tire’s working conditions on the estimated parameters.

The nomenclature used to refer to the key points or parameters of the strain curves is shown in Figure 5. The maximum tensile strain values are denoted as $\varepsilon_{x}$ and are indicated by green squares on the strain curve, while on the time derivative curve, maximum peaks are indicated as $d\varepsilon_{x}$ and shown by purple triangles.

It can be seen that the strain curve in the circumferential direction and its time derivative exhibit similar behavior as described in the literature [34,35,48]. The strain wave morphology is characterized by a maximum peak (tensile strain) and two minimum peaks (compression strain) in the circumferential direction for a tire rolling conditions. While, in the waveform of the time derivative of strain, a maximum peak at tensile and a minimum peak at compression can be differentiated. In the circumferential strain curve, the tensile strain takes place within the tire-contact length where at its center registers the peak of maximum strain denoted as $\varepsilon_{x}$. On the contrary, the peaks in compression are located at outside of the contact patch, and are the points where the deformation toward outside of a tire is the maximum by loading [13,34,35,36,48].

**Figure 6:**
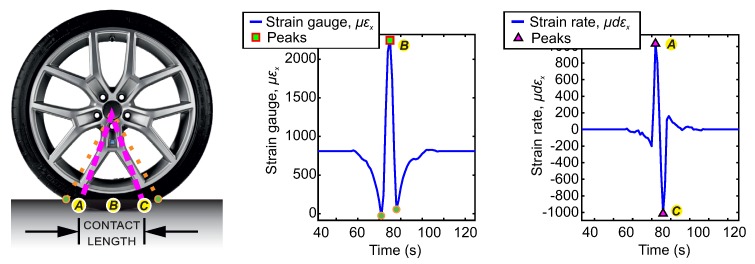
Characteristics of the contact trace on the circumferential strain curve ($\varepsilon_{x}$) and on the derived strain curve ($d\varepsilon_{x}$). Operating conditions: 0.8 bar, 0°, 50 km/h, 500 N.

In order to analyse the deformation curve in the circumferential direction experienced by a point in the tire-contact patch, Figure 6 shows the waveform of the circumferential strain curve and its time derivative. It illustrates the relation between the strain parameters and the tire parameters. This point contains the strain sensor (length of sensor—2 mm).

The deformation sensor registers the offset or displacement of the curve when it is located outside of the ground contact area. The green circles point the boundaries of the deflection (contact) zone. Within the deflection (contact) zone is the bonding zone which defines the contact length. The sensor registers a compression strain before entering and after leaving the contact patch. When this sensor is inside the contact patch, a tensile strain is observed. In the green circles (maximum compression peaks in the strain curve, $\mu \varepsilon_{x}$), the tread deformation towards outside of contact maximizes, just before and after the contact patch. This is due to the effect of the vertical load on the tire. Within the contact patch, the point “B” (peaks of maximum tensile) becomes dominant due to the bending of the tread by the effect of vertical loading [12].

In the time derivative of the strain curve, the points “A” and “C”, indicate the trailing and leading edges of the contact patch. The distance between them is an approximation of the contact length [48] which also, defines the length of the area where the phenomenon of adhesion occurs [50].

Morinaga et al. [48] defines the distance between the maximum compression peaks of the strain waveform in the circumferential direction (green circles in the Figure 6) as *deflection length*, since this area of the tire is bent. Similarly, the distance between the maximum and minimum peaks (points “A” and “C”), in the waveform of its time derivative, just where the change in deformation is maximized, is referred to as the *contact length*. That is, when the tread elements enter or leave the contact patch, the deformation at a certain point changes from the contact length to the deflection length, generating a sudden variation from the tensile to the compressive deformation and viceversa.

Additionally, Kim et al. [35] study the influence of vertical load, slip angle, and wheel velocity on the strain curve in the circumferential direction and its time derivative. They point out in their study that the maximum peak in the deformation curve and, the distance between the peaks of the derivative curve increases as the vertical load increases. This trend coincides with the physical phenomenon that the greater the vertical load, the greater the length of contact in the contact length. Furthermore, he points out in his study that, the maximum tensile peak in the strain curve decreases gradually with increasing longitudinal speed. That is, without significantly affecting the distance between the peaks in the strain curve. In the variation of the tire slip angle, no correlation with the maximum tensile peak of the strain curve was observed, nor is it defined how this variation affects the distance between maximum peaks of the time derivative strain curve. However, research has shown that the shape of the contact length changes under the influence of lateral force [12,34,48] and consequently the length of the contact patch changes.

### 3.2. Development of the Algorithm

The instrumented tire consists of three rosettes set up to the inner surface of the tread band. In the Section 2.2 it is explained that two of the three strain gauge sensors measure in the axial direction (channel 1 and 3) and, the last sensor measures in the circumferential direction (channel 2). Therefore, if the main direction of the strain gauge is parallel to the wheel plane, then it is defined as strains in the circumferential direction. On the contrary, if the measuring direction of the strain gauge is parallel to the transverse plane of the wheel, these strains are defined as axial strains.

Simultaneous longitudinal and lateral slip, the tire is subject to a carcass lateral deflection, which changes the main measuring direction of the strain sensors. Strain sensors on the inner surface of the tread measure strain of a rolling tire in the contact patch.

**Figure 7:**
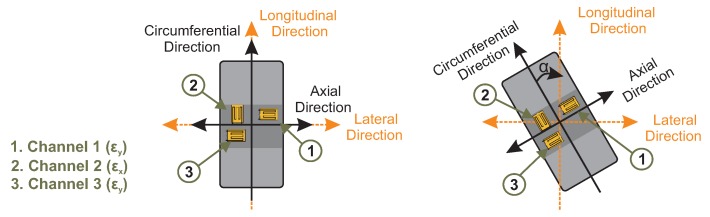
Main measuring direction of the strain sensors.

Two coordinated systems are illustrated in Figure 7, the first one fixed concerning the vehicle reference system and indicated in the figure as longitudinal and lateral direction. The second system particularizes circumferential and axial axes in the longitudinal and lateral wheel planes. The latter specifies the main measuring direction of the strain sensor. Pure rolling, both reference systems coincide while, in pure and combined sideways sliding, they differ.

This methodology studies the strains in the circumferential direction, i.e., the strain curves recorded by channel 2. Its main measurement direction is parallel to the wheel plane, so this curve describes the strain along the contact patch.

**Figure 8:**
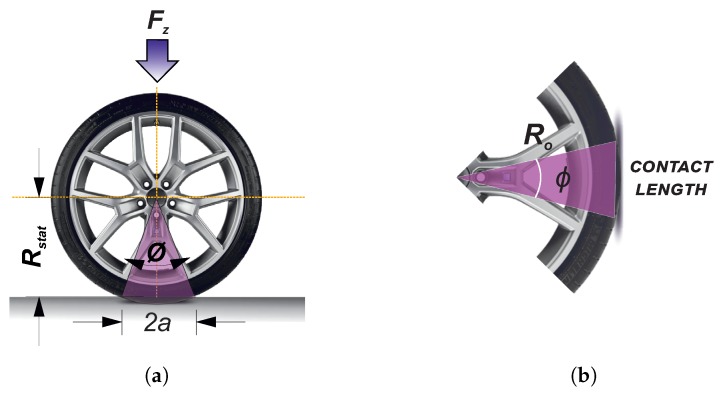
Relationship between contact length and effective rolling radius. (**a**) Calculation of the effective radius according to Rajamani; (**b**) Estimation of the contact length.

Rajamani [51] states that the linear equivalent of the rotational speed of the tire ($v_{eff}$) is the product of the effective radius ($R_{eff}$) and the angular velocity of the tire ($w_{w}$). Further, it points this is equivalent to the ratio between the longitudinal length of the contact patch, 2a, and the time, *t*, taken by an element of the tire to move through the contact patch (see Figure 8b).

(1) $$v_{eff}=R_{eff}\cdot w_{w}=\frac{a}{t}$$

Figure 8a shows a representations of the relationship between the $cl_{w}$ and the $R_{eff}$, nonetheless, $R_{eff}$ is a parameter is related to the static radius ($R_{stat}$) and the nominal or undeformed radius of the tire ($R_{0}$), according to the following relation $R_{stat}<R_{eff}<R_{o}$ [51].

The longitudinal distance of the contact patch is determined from the time derivative of strain of channel 2 [48]. Lee et al. [12] explain the distinctive peaks on its waveform indicate where the change of strain is maximized because when tread elements enter or leave the contact patch, the strain at the corresponding point on inner liner experiences sudden change. The tensile strain takes place within the contact patch. Thus, it is accepted that tensile strain due to the adhesion of the tread elements at the leading edge and the trailing edge of the contact patch are indicated by the maximum peaks of the time rate of the strain waveform.

**Figure 9:**
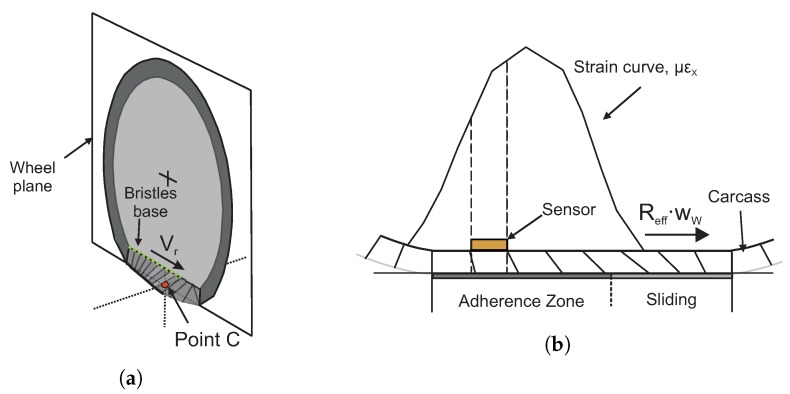
Hyphoteses of the strain measurement. (**a**) Example of the velocity in the base point of the bristle; (**b**) Ilustrations of the strain measurement for a tire bristles in the adhesion zone.

According to Pacejka [52], the brush model explains the interaction of the tire tread elements, called as bristles, with the road surface. It explains that when the tire rolls the bristles experience a deflection in a direction parallel to the road surface where the tread elements move from the leading edge to the trailing edge. A hypothetical situation is to assume the strain curve of the measurement point (where the strain sensor is placed) as the strain suffered by a tip element of the tread band, since the strain measure is taken at the base of a bristle. The tip remains adhered to the ground under the condition of the available friction. Simultaneously, the base point of the bristle remains in the wheel plane and moves backward with the linear speed of rolling $V_{r}$ with respect to the contact center C (see Figure 9). However, the bristle’s base, with respect to the road, moves with a velocity that is designated as the slip speed $V_{s}$ of the wheel.

Based on the literature [48,52], the measurement point describes the strain experienced by an element of the tread when it passes through the contact patch. The strain curve (channel 2) is referenced to the wheel plane thus, the strain measure along the contact patch. The contact lengths, $cl_{w}$, can be estimated from peak to peak distances of the strain rate [12,48]. The linear speed estimated from the strain curve in the circumferential direction is assumed to be the velocity of a bristle passing through the contact length.

The tire is an element that forms the chord of a circular segment when it comes into contact with the road. In the Figure 8, this chord is shown which is defined as the contact area of the tire. The relationship between the contact length, $cl_{w}$, and the central angle, formed by the radial line joining the center of the wheel with the ends of the contact patch is indicated as ϕ. The central angle, ϕ, is calculated through the strain curve. This reasoning allows for obtaining a quantifiable value from the length of the contact patch. Similarly, through this angle the effective radius of the tire can be estimated, $R_{eff}$.

**Figure 10:**
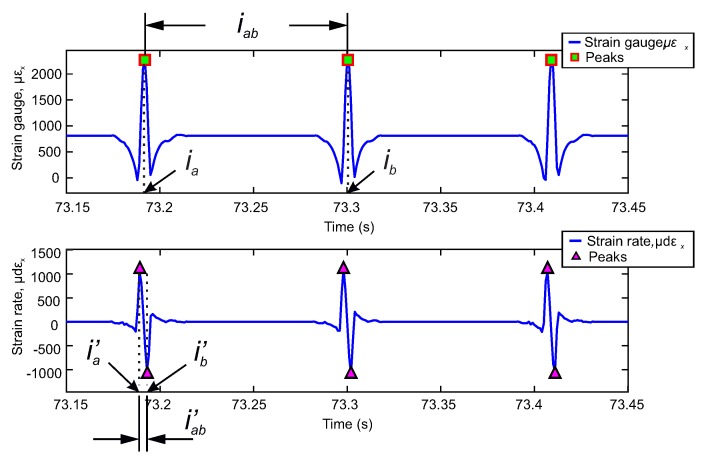
Calculation of the contact length using the strain curve in the circumferential direction (0.8 bar, 0°, 50 km/h, 500 N).

Therefore, the linear equivalent of the tire’s rotational speed can be computed by the ratio of the contact length and the time it takes for an element to move through it. The time that elapses when a point passes through the contact length can be calculated from the time derivative of the strain curve (see Figure 10). In this study it is assumed that the distance from peak to peak in the strain rate curve is equivalent to the time requested in Rajamani [51].

Therefore, to estimate the contact length in the circumferential direction, $cl_{w}=2a$, the following considerations are made:

- The distance between the maximum peaks in the strain curve at circumferential direction, “$i_{ab}$”, describes a rotation angle of approximately 360° (2 π·rad).
- The time lapse between the maximum and minimum peaks in the time derivative strain curve in the circumferential direction, “$i_{ab}^{\prime }$”, is equivalent to a time *t*.

Herein it is assumed that the contact length operates on a circular segment, where the endpoints are equidistant from the wheel center (see Figure 8b). The central angle, ϕ, is computed across the strain curve in degrees or radians using the following relationship:

(2) $$\phi =\left(2\pi \cdot rad\right)\cdot \frac{i_{ab}^{\prime }}{i_{ab}}=\left(360\right)\cdot \frac{i_{ab}^{\prime }}{i_{ab}}.$$

The distance between peaks (maximum and minimum peaks) in the time derivative of the strain curve is expressed on the time axis (time(s)). However, previous research [36], shows the distance between strain peaks by the angle of rotation. Therefore, according to the procedure followed in this study, if the distance between peaks in the time derivative of the strain curve in the circumferential direction is expressed as a function of a time *t*, it must be converted into angle (radians or degrees) using the Equation (2).

Finally, by adapting the proposed estimation process to the Rajamani concept, the linear speed of the wheel in the contact patch can be estimated, $V_{w}$. The equations to be used in this study to calculate the requested tire parameters are described below. The definitions of the abbreviations are described in an abbreviations’ list at the end of the document.

(3) $$cl_{w}=2\cdot R_{o}\cdot \sin(\frac{\phi }{2})$$

(4) $$R_{eff}=\frac{cl_{w}}{\phi }$$

(5) $$\omega_{w}=\frac{2\cdot \pi \cdot F_{s}}{i_{ab}}$$

(6) $$V_{w}=\frac{cl_{w}}{t}$$

Other dynamic parameters of the tire, i.e., $R_{stat}$, $k_{t}$; can be estimated with the strain measurements obtained with the intelligent tire. However, in this research, they are irrelevant for the moment.

## 4. Computed Experimental Results

In the Section 3.2, the proposed methodology for calculating the tire parameters ($R_{eff}$, $cl_{w}$ and $V_{w}$) has been indicated. The experimental data obtained with the instrumented tire is used to implement this procedure. Based on the results, this section discusses the influence of operating conditions on these parameters. The analysis made about the estimated parameters applies within the boundaries of the experimental test (most important limits for the conditions of a formula student car) and this analysis should be checked for any data outside of those boundaries.

The results presented the tire operational conditions of the experimental tests ($V_{x}$, $F_{z}$, and α) as the independent variables and the tire estimated parameters ($R_{eff}$, $cl_{w}$ and $V_{w}$) as the dependent variables. Further, the figures of the contact length and the effective radius are shown into two forms, (a) and (b), to simultaneously evaluate the influence of speed and vertical load on the estimated parameters.

**Figure 11:**
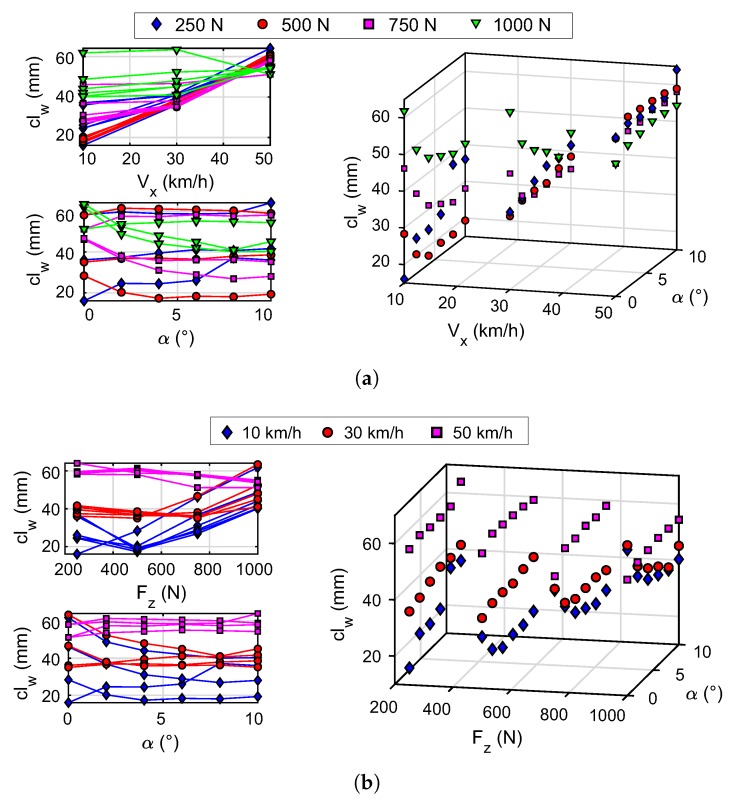
Contact patch results obtained by applying the proposed methodology to the experimental data. (**a**) Data differentiated by vertical load; (**b**) Data differentiated by rolling speed.

Figure 11a shows the results of $cl_{w}$ as function of $V_{x}$ and α, and the data were classified by colors according to $F_{z}$ values. It is observed that the length of the contact patch ($cl_{w}$) converges to a value as the speed of the wheel increases from 10 km/h to 50 km/h. This phenomenon is more evident for vertical force values below 1000 N. For the maximum test speed (50 km/h) the value to which this parameter converges is limited between 50 mm and 60 mm. Herein can be observed that the contact length has a high dependence on speed.

The influence of the vertical load and the slip angle is less at higher wheel speeds. The influence of the vertical load shows the layered points, and with a convergent trend as the slip angle increases. This trend is more pronounced at low speeds and at higher vertical loads. Specifically, the contact length tends to decrease as the slip angle increases for speed values below 30 km/h and vertical load values above 500 N.

Figure 11b illustrates the results of $cl_{w}$ with $F_{z}$ and α as the dependent axes and, the data colored is in line with $V_{x}$. This shows that the increase in vertical load also produces a convergence of the contact length to a constant value. This parameter converges to a value between 40 mm and 60 mm for a vertical load value of 1000 N. At lower speeds the influence of the vertical load is greater; this influence is lower at higher speeds, i.e., at 30 km/h and 50 km/h. In addition, it is evident that speed stratifies the contact length values independently of the values of $F_{z}$ and α. These layers (stratification) tend to converge to the value mentioned above. About the slip angle, it is observed that its influence on the contact length is smaller at higher speed.

The relationship between the contact length and the slip angle, within the range of experimental values studied, depends on the speed and the vertical load applied to the tire. At low speed, the influence of the slip angle on the contact patch is greater for greater vertical load. On the other hand, at higher speeds, the behavior of the curves is less pronounced, with less variability in the contact length results.

**Figure 12:**
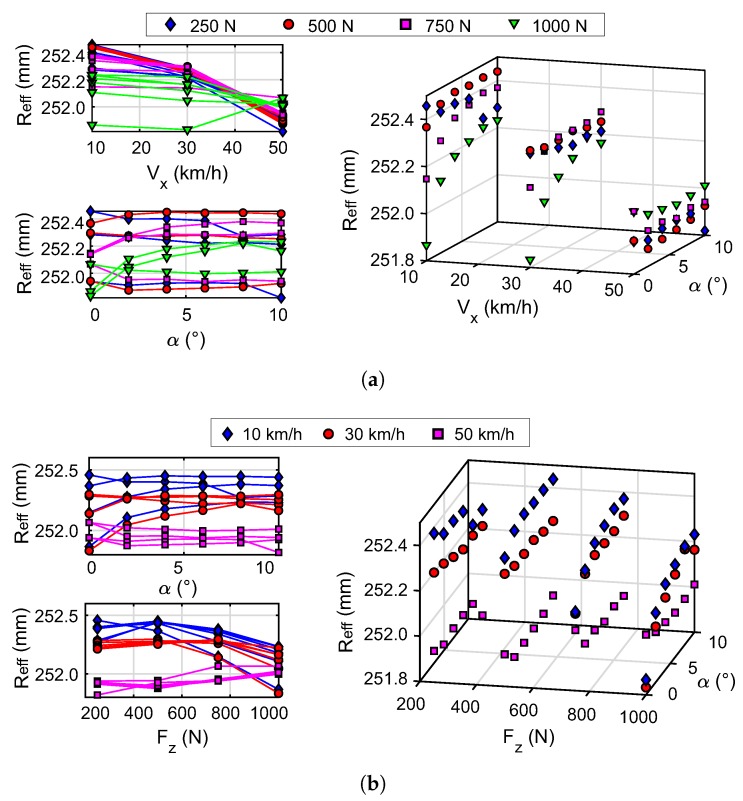
Effective radius results obtained by applying the proposed methodology to the experimental data. (**a**) Data differentiated by vertical load; (**b**) Data differentiated by rolling speed.

Figure 12 shows that $R_{eff}$ has a similar but inverse behavior to that shown by $cl_{w}$. Thus, the analysis of Figure 11 can apply to Figure 12. The results of the effective radius for the conditions tested are shown in Figure 12a. Similarly to the contact length, the magnitude of the effective radius converges to closer values when increasing the speed of the wheel. For the tested speed of 50 km/h this parameter tends to a value between 251.8 mm and 252.1 mm. It can be seen that the increase in the slip angle reproduces the trend to a constant value, which is highly varying or more abrupt with increasing vertical load. This behavior is clearly seen at speeds of 10 km/h and 30 km/h. Figure 12b shows how the speed stratifies the values of the effective radius. The layers (stratification) observed in the effective radius converge to values between 251.8 mm and 252.2 mm for 1000 N.

The inverse relationship noticed between $cl_{w}$ y $R_{eff}$ in Figure 11 and Figure 12 agrees with Matsuzaki and Todoroki [36]; the increase in contact length indicates greater deformation in the tire, the center of the tire becomes lower, resulting in a reduction of the effective radius. In these figures it can be seen that the contact length and effective radius vary, from 60 mm to 48 mm and from 251.8 mm to 252.2 mm respectively, by increasing the speed from 10 km/h to 50 km/h for a vertical load of 1000 N and a slip angle of 0°.

The results obtained show consistency since stability is observed when the experimental conditions vary. It is checked that the contact length and effective radius depend on the wheel speed, vertical load, and slip angle.

Values with a less clear trend are observed for a vertical load of 250 N, so they seem to be associated with a higher tire slippage. This dispersion of data is further accentuated at lower speeds, e.g., at 10 km/h.

Xia [53] explains that the increase in speed, making the tire roll faster, reduces the vertical load time on the tire. This can explain the proximity of the values of the effective radius and the contact length to different vertical loads when increasing the speed to 50 km/h (see Figure 11a and Figure 12a).

The convergence observed in the parameters studied when varying the slip angle is related to the brush model [52]. The increase in the slip angle means that the length of adhesion tends to a constant value called the pure lateral slip state. However, it is interesting to study in detail the trend of the contact length curves when the slip angle varies (within the range of experimental values studied). In this study was observed that the variable that most influences the contact length and the effective radius is the speed of the wheel.

The procedure proposed in Section 3 allows estimating the linear speed of the wheel in the tire contact patch as the velocity of the strain gauge when crosses the contact length. The strain gauge on the inner surface of the tire detects the strain to which this point is subjected. As the slip angle increases, the circumferential axis of the wheel changes its direction relative to the longitudinal axis of the vehicle. Therefore, the strains are measured in the middle plane of the wheel. This allows computing the circumferential speed of the tire ($V_{w}$), as the speed at contact point *C*. This procedure is easy to implement, without requiring complex algorithms or additional equipment to the tire instrumentation to obtain this parameter [54].

**Figure 13:**
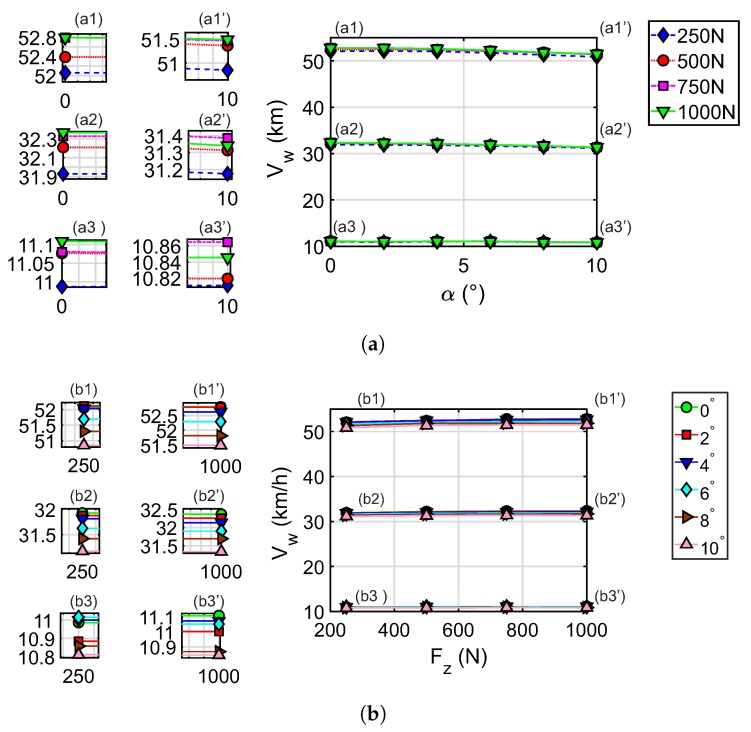
Results of the wheel speed in the contact footprint obtained by applying the proposed methodology to the experimental data.(**a**) Data differentiated by vertical load; (**b**) Data differentiated by slip angle.

Figure 13 is composed of two graphs Figure 13a,b, one shows the relation between $V_{w}$ and α whose curves represent the how it is influenced by $F_{z}$ as indicated by the legend and, the other shows the relation of $V_{w}$ and $F_{z}$ whose curves represent the α values. In both graphs, it can be seen that at lower speeds the curves tend to overlap on the same line. In the relationship between speed and slip angle, it can be seen that increasing the slip angle slightly reduces the speed values. Figure 13a illustrates how the velocity changes from 52.8 km/h at 0° to 51.5 km/h at 10°, similar situation happens at lower velocities. Furthermore, Figure 13b shows that $V_{w}$ decreased when α increased. This is due to the tire slippage. The relationship between speed and vertical load has a growing trend. This is because the vertical load favors the traction of the wheel. Less dispersion is observed in the vertical load curves, indicating that the slip angle has more influence on the speed transmitted by the wheel.

**Figure 14:**
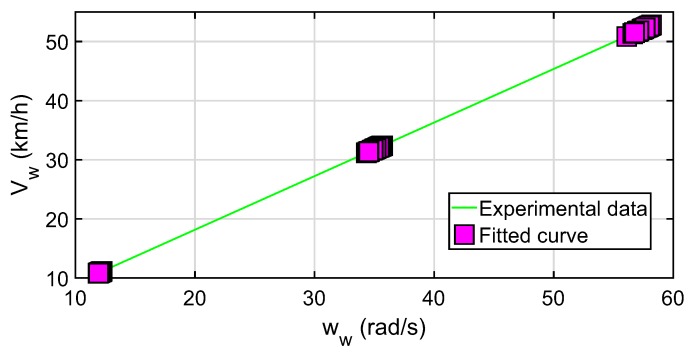
Relationship between tire speed, $V_{w}$, and rotation speed, $\omega_{w}$, calculated through the methodology proposed in the experimental data.

As explained above, Equation (5) is used to estimate the angular velocity of the tire from the strains measured in the experimental tests (also see Yang et al. [34,55]). To evaluate the results obtained from the speed of the wheel at the point of contact, $V_{w}$, the correlation of this with the angular speed of the tire, $\omega_{w}$, is made by means of a least-squares adjustment. Figure 14 shows that the setting (green line) is positive. The degree of correlation of the points of the wheel speed (purple square points) fits perfectly with the model.

It is interesting to note that the increase in drum’s speed in the experimental tests produces greater variability in the linear and angular speed of the tire. This is shown by the scattering of the purple square dots. Therefore, it corroborates that both the vertical load and the slip angle influence (slightly) the magnitude of the velocity, as observed in Figure 13. The spread observed is the result of the tire slippage that occurs when the working conditions of the tire change. As the tire speed increases, the strain measured in the contact patch is more sensitive to changes in the slip angle and vertical load.

Calculating the speed of a point at the tread band within the contact patch provides information on the tire’s slip. Slippage is related to the tire’s ability to adhere. Less dispersion in the values of the contact length and the effective radius was observed when increasing the speed and vertical load on the tire.

The behavior observed in the contact length and the effective radius show the variability in the dynamic behavior of the tire, highlighting the high complexity involved in the study of the tire. Therefore, monitoring these parameters while the tire rolls make it possible to obtain real-time information on the interaction of the tire with the surface. This allows for the optimization of existing vehicle stability control systems.

Therefore, the procedure developed in this study allows complementing the methodology developed in [28]. These parameters are fundamental to the development of a methodology for detecting slippage through the strain measured in the tire.

These results demonstrate the ability of the instrumented tire with strain sensors to detect speed in the contact patch, effective radius, and contact length. This emphasizes the need to go deeper into these studies, since the references found on the influence of operating conditions on these parameters are limited. From this analysis, the inputs to be used to develop an estimator of the effective radius and contact length in complex maneuvers are selected.

## 5. Tire Parameters Estimator

This section discusses the development of an estimator of the effective radius and length of the contact patch under demanding dynamic maneuvering conditions. The purpose is to estimate these tire parameters under different running conditions, as well as to monitor the tire as it rolls on the road.

The heuristic rules of the estimator are based on the results of the algorithm applied to the experimental data. The purpose is to extrapolate the results to other types of working conditions, enabling the evaluation of their behavior in complex dynamic maneuvers. CarSim software simulations are used to obtain the input data for the estimator under severe maneuvering conditions.

**Figure 15:**
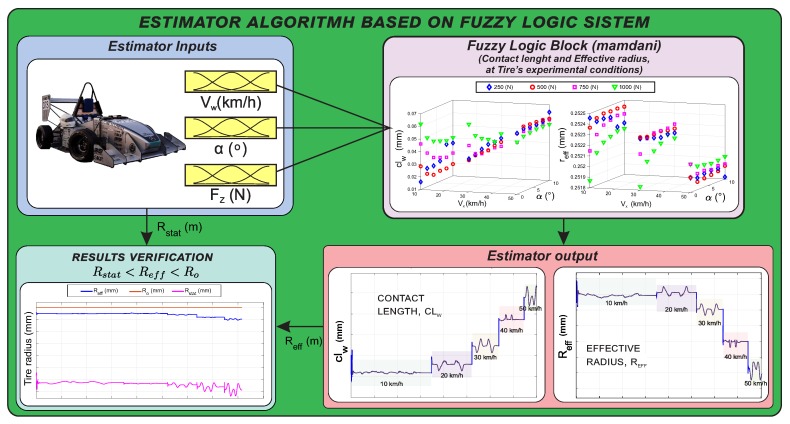
Algorithm of the development of the fuzzy logic estimator.

This estimator enables the results of the developed algorithm to be verified due to the estimator is based on those results. If the results of the fuzzy logic estimator are consistent under dynamic maneuvering conditions (see Figure 15), this would indicate the validity of the algorithm proposed in this document.

**Figure 16:**
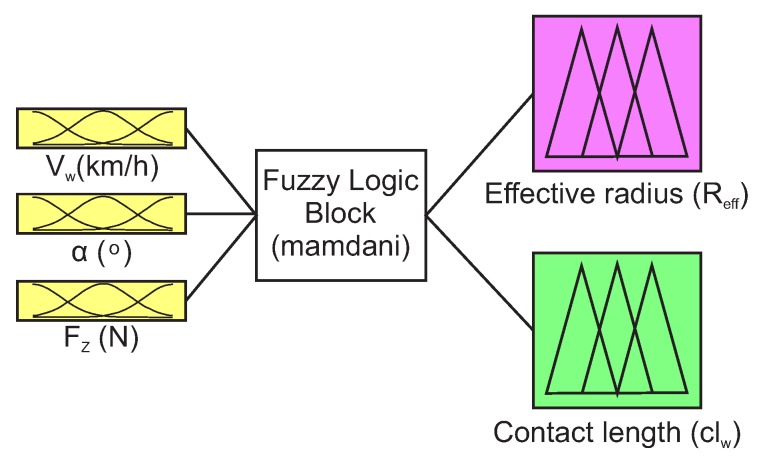
Estimator architecture developed through fuzzy logic.

For the fuzzy logic system, it has been considered to feed the system with the slip angle, the vertical load and the wheel speed at the contact patch, given their influence on these parameters (see Section 4). The inputs to the fuzzy logic system can be obtained directly through the strain measurements, as shown in [28] and the Section 3; to test the effectiveness of the estimator, the simulation software CarSim has been used to obtain these parameters under the requested conditions. Figure 16 shows the scheme of the developed estimator, the inputs to the fuzzy block and the output parameters (contact length and effective radius) being specified.

CarSim is a commercial software that accurately simulates vehicle behavior through mathematical models. The software is an industry standard, being used by car manufacturers (General Motors, Toyota, Honda, Ford, etc.), suppliers, and research laboratories and universities [9,54,56,57,58,59,60,61,62,63,64,65,66]. This software contains the main effects that determine how the tire contacts the road and how the forces are transferred in the tire–road interaction through the suspension to the chassis. However, they do not have details of link connections or structure compliance, nor do they provide information on the behavior of the effective radius and contact length during the simulation.

In order to obtain the data of the input variables under the dynamic maneuvers, the Formula 3 vehicle configuration (F3) was set up to a formula student car, thus test conditions are better suited to the type of tire tested under real operating conditions.

**Figure 17:**
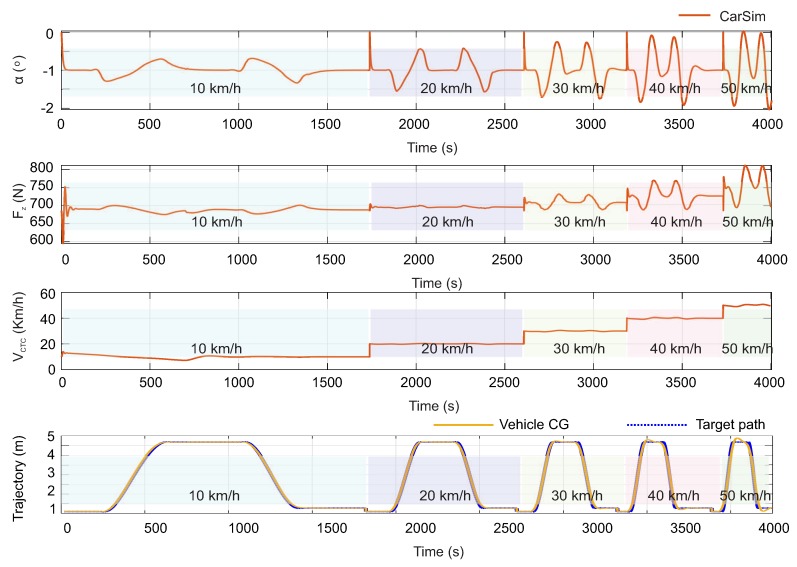
Inputs variables (CarSim variables - wheel L1) to test the estimator at complex maneuvers.

In this section, the double lane change (DLC) test is used at the speeds of 10 km/h, 20 km/h, 30 km/h, 40 km/h and 50 km/h. Figure 17 shows the fuzzy logic system inputs. These are extracted from the CarSim software for the L1 wheel: slip angle, α, vertical load, $F_{z}$, tire speed in the contact patch, $V_{CTC}$.

The trajectories developed in the simulation tests are illustrated in the last graphic of Figure 17 and they show the severity of the maneuver. Herein is shown the target path and the trajectory described by the center of gravity of the vehicle (Vehicle CG). In each chart is indicated the speed at which the test is performed (in the same timeline are depicted five tests for five different speeds). On the other hand, in the figure, it can be observed that the values of the simulated inputs are within the range of values tested with the instrumented tire since the fuzzy logic estimator has been designed from such experimental data.

**Figure 18:**
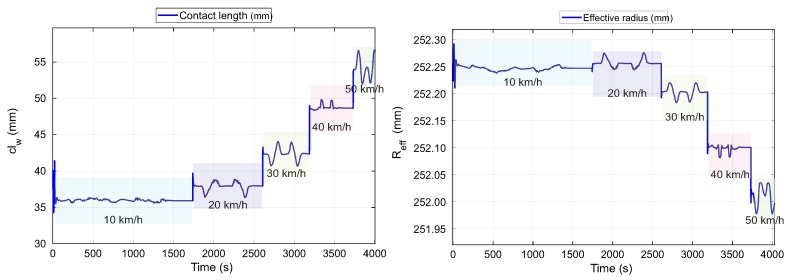
Results of fuzzy logic estimator, effective radius, $R_{eff}$, and contact length, $cl_{w}$.

The results of the contact length and the effective radius estimated by the fuzzy logic system are shown in Figure 18. These graphics show the behavior of tire parameters at the indicated maneuvers. It can be noticed that by varying the speed of the wheel from 10 km/h to 50 km/h, the contact length increases while the effective radius decreases. Likewise, it is emphasized that at 10 km/h the variability shown by the effective radius and contact length is less than for the rest of the simulated speeds.

The variability observed in the results of the effective radius and in the contact length at 10 km/h coincides with the experimental behavior since at this speed the influence of the slip angle was low (see Figure 11 and Figure 12). At 40 km/h the curves of these parameters ($cl_{w}$ and $R_{eff}$) are less abrupt. A lower influence of vertical load and slip angle is observed. At 50 km/h the curves of $cl_{w}$ and $R_{eff}$ are inverse to those obtained at previous speeds. The behavior observed coincides with the results shown in the Section 4. Speed proved to be a crucial variable in the behavior of these parameters, where their tendency, by varying convergence and vertical load, is affected.

**Figure 19:**
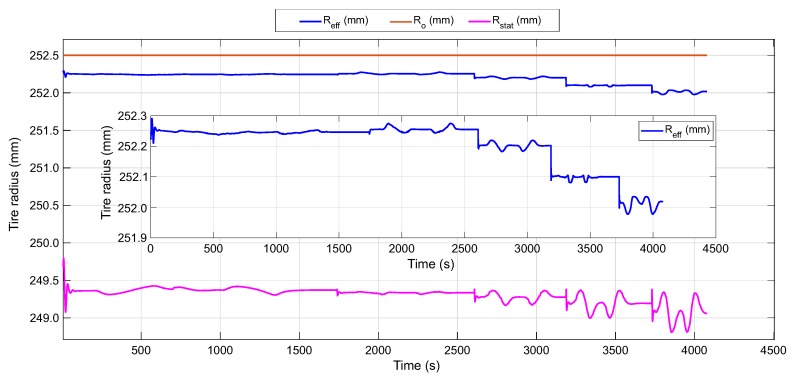
Comparison between the estimated effective radius, $R_{eff}$, the static radius, $R_{stat}$, and the undeformed radius, $R_{o}$, of the tire under the DLC maneuver at different speeds.

Since the static radius, $R_{stat}$, can be extracted from the CarSim simulation software for the set maneuver, the result of the effective radius of the proposed estimator is contrasted in Figure 19 with the undeformed radius, $R_{o}$, and the static radius, $R_{stat}$, of the tire. The static radius describes the position of the wheel center. Rajamani [51] refers to the following relation $R_{stat}<R_{eff}<R_{o}$, indicating that this condition should be met. In addition, in radial tires, the rolling radius is closer to the undeformed radius than the static radius [57].

The result is consistent with the conditions expressed by the authors [51,57]. There is similarity between the effective radius, $R_{eff}$, and the undeformed radius, $R_{o}$, as well as, the estimated $R_{eff}$ is within the limits proposed. Although the values of $R_{o}$ and $R_{stat}$ are from a simulation, the study of their variations throughout the analysed maneuver shows great similarities.

It can be noticed how the CarSim results at 50 km/h do not show that change in direction of the static radius curve, $R_{stat}$, nor the decrease in magnitude of the effective radius at 40 km/h. Therefore, it is considered important to further explore the influence of speed and vertical load on the contact length and effective radius.

## 6. Discussion

Previous studies have pointed out the importance of estimating the rolling conditions of the tire to optimize the vehicle’s control systems. The effective radius, the length of the contact patch, and the speed of the tire are parameters linked to tire slip. Besides, they are related to the friction models of the tire for estimating the conditions of the tire–road contact surface during driving. The initial objective of this study is to propose a methodology to obtain these parameters ($cl_{w}$, $R_{eff}$ and $V_{w}$) from the strains measured on the contact patch.

Experimental data obtained under controlled operating conditions are used to verify the proposed methodology. The results obtained from the effective radius and contact length, through the experimental data, show consistency with the variation of the test conditions. Both parameters describe an inverse relationship, the instantaneous center of rotation approaches the geometric center of the wheel, resulting in a reduction of the effective radius and an increase in the contact length of the tread. This coincides with the studies conducted by Matsuzaki and Todoroki [36].

Within the analysed experimental range, wheel speed is one of the variables that most influence the dynamic behavior of the tire parameters ($cl_{w}$ and $R_{eff}$). As observed in Figure 11 and Figure 12, as the wheel speed increases, the influence of the vertical load and the slip angle is decreased.

The contact length converges to a finite value as the slip angle increases. This agrees with Pacejka’s brush model under the effect of pure sliding. However, the experimental results show that the trend of the contact length curves when varying the slip angle is related to the speed and vertical load of the wheel, as shown in Figure 11. Additionally, the speed and vertical load stratify the curves of this parameter. The higher the speed, the less dispersion is shown in the contact length curves as the vertical load and the slip angle vary (see Figure 11b). The curves are abrupt at higher vertical loads and lower speeds.

The effective radius yields similar results to the contact length since the relationship between them is inverse. Under the conditions tested, the effective radius converges to a finite value as the slip angle increases. The trend of these curves is mainly affected by the speed of the wheel. It can be seen that the curves are stratified for each speed, with the curves being less dispersed as this variable increases (see Figure 12b). The vertical load on the tire also affects the degree of variation in the curves.

At 250*N* a less clear trend was observed in the curves of the parameters studied ($cl_{w}$ and $R_{eff}$) as a result of the low vertical load on the tire.

The speed of the wheel is computed over the tire–road surface. It is the speed of the wheel point where the strain sensor is fixed when it passes through the contact patch. Figure 13 shows the result of this speed as a function of the tire operating variables. Greater variability in wheel speed was observed in response to the variation in slip angle and vertical load. This variability is also seen in the adjustment made between the tire speed, $V_{w}$, and the angular speed, $w_{w}$, in Figure 14. The correlation between the two is linear.

It has been observed that the speed of the tire, $V_{w}$, should be less than the speed of the drum, $V_{x}$. However, this is attached to an increase in the speed of the drum due to its inertia, which cannot be detected by the speed measurement implemented in the test system. This difference is considered not significant for the study carried out, given that the speed of the drum is used as a guiding variable.

The tire is a complex component to analyse since it is difficult to accurately predict its response under different working conditions. In the study carried out, interesting relationships were observed that had not been considered when evaluating this component, such as the influence of speed, vertical load and drift angle on $cl_{w}$ and $R_{eff}$. This subject requires further study since this work has shown the behavior of the tire’s parameters over a range of speed and vertical load.

Additionally, with the results of $cl_{w}$ and $R_{eff}$ in the test conditions a fuzzy logic estimator was developed. This allows us to check the results of the parameters under different dynamic conditions. The variables $F_{z}$, $V_{ct}$, and α are used as input data. This data is obtained from the CarSim software under a Formula Student vehicle configuration in a DLC maneuver at different speeds. The results of the estimator show coherence with the established maneuvers, this provides sense to the algorithm proposed in this study. The effective radius and the contact length during the proposed dynamic maneuvers show the opposite behavior between them. Further, the condition between the effective radius ($R_{eff}$), the undeformed radius of the tire ($R_{o}$) and the position of the center of the wheel ($R_{stat}$) is verified in Figure 19.

## 7. Conclusions

A tire instrumented with strain gauges was tested at the vehicle laboratory of the University of Birmingham. The trials were conducted under controlled operating conditions. This study proposes a methodology that uses these experimental data to estimate the effective radius ($R_{eff}$), the length of the contact patch ($cl_{w}$), and the speed of the wheel at the point of contact ($V_{w}$). The proposed methodology allows the determination of these parameters without requiring complex algorithms for their estimation, as well as the instrumentation of the vehicle to obtain additional variables. The results given represent an approximation of the tire parameters ($cl_{w}$, $R_{eff}$, $V_{w}$) under controlled conditions allowing the evaluation of their behaviour under controlled conditions.

The present study shows that wheel speed is a crucial variable in the dynamic behaviour of the tire. The contact length and the effective radius of the tire converge to a limit value as the slip angle increases. However, the speed affects the trend (increasing/decreasing) of the curves that relate these parameters to the slip angle. It was also observed that speed and vertical load on the tire stratify both parameters ($cl_{w}$ and $R_{eff}$), with the layers being more accentuated in the case of speed. It was also observed that when the vertical load on the tire increases, the curves are abrupt. However, the effect of the slip angle and vertical load is reduced as the speed increases. It is interesting to observe this behavior since the tire is characterized by a critical speed at which circumferential waves begin to act and increase friction. However, it is proposed to carry out a more detailed study on the influence of speed and vertical load on this behaviour. It is evident that the effective radius and the contact length have an inverse relationship, concluding that there is a close relationship between both.

Based on the brush model, the speed of one of the bristles in the tire contact patch ($V_{w}$) can be estimated through this methodology. The results of $V_{w}$ indicate that, with increasing speed, the effects of slip angle and vertical load are perceived.

The analysis of the parameters obtained under controlled test conditions is used to form a fuzzy logic estimator. The fundamental input variables are vertical load, speed, and slip angle. The estimator developed in fuzzy logic enables the verification of the results obtained in controlled conditions by applying them in complex maneuvers.

Considerable progress has been made with the methodology proposed to estimate the tire parameters related to the tire–road interaction. Their estimations at controlled operating condition provide an overview of the contact patch and enables to understand the influence of the tire working condition in these dynamic parameters. As an advantage, this methodology only requires the tire strain measurements, therefore, it can be applied to any tire. Nonetheless, this should be checked in further studies.

It would be convenient to address in future works the implementation of this methodology to the strains measured at different operating conditions from the ones shown here. This research establishes the basis for a friction estimation system based on the information recorded by an intelligent tire.

## Abbreviations

The following abbreviations are used in this manuscript:

MDPI	Multidisciplinary Digital Publishing Institute
DOAJ	Directory of open access journals
TLA	Three letter acronym
LD	linear dichroism
ABS	Anti-lock Braking Systems
EBD	Electronic Braking Distribution
SAW	Surface Acoustic Wave
IDT	Interdigital Transducer
FBG	Fiber Bragg Grating
MEMS	Micro Electro Mechanical Systems
GPS	Global positioning system
FS	Formula Student
$R_{eff}$	Effective radius at the contact patch
$R_{o}$	Undeformed tire radius
$R_{stat}$	Static radius
$k_{t}$	vertical stiffness
$F_{s}$	Sampling frequency
$cl_{w}$	Contact length at the contact patch
$V_{w}$	Wheel velocity at the contact patch
$v_{eff}$	Linear equivalent of the rotational speed
$V_{x}$	Linear drum’s rolling speeds
α	Slip angle
$F_{z}$	Vertical load
Φ	Central angle
$\omega_{w}$	Angular velocity of the wheel
DLC	Double lane change
CG	Centro de gravedad
